# Excessive dietary lead reduces growth performance and increases lead accumulation in pigs

**DOI:** 10.5713/ajas.20.0220

**Published:** 2020-07-02

**Authors:** Hyunjun Choi, Sang Yun Ji, Hyunwoong Jo, Minho Song, Beob Gyun Kim

**Affiliations:** 1 Department of Animal Science and Technology, Konkuk University, Seoul 05029, Korea; 2 Animal Nutritional Physiology Team, National Institute of Animal Science, Rural Development Administration, Wanju 55365, Korea; 3 Department of Animal Science and Biotechnology, Chungnam National University, Daejeon 305764, Korea

**Keywords:** Exposure Time, Lead Accumulation, Organ, Swine, Tissue, Toxicity

## Abstract

**Objective:**

The objective of this study was to investigate the influence of dietary lead (Pb) supplementation and feeding period on growth performance, organ weight, and Pb accumulation in pigs.

**Methods:**

In a 56-day feeding experiment, a total of 48 barrows with initial body weight 10.4±0.6 kg were allotted to 2 dietary treatments (0 and 200 mg/kg of supplemental Pb) in a completely randomized design with 6 replicates. Body weight and feed intake were recorded to calculate growth performance. At the end of each 14 day-period (on days 14, 28, 42, and 56), an animal was randomly selected from each pen and slaughtered to collect blood samples, hair samples, left 5th rib, heart, liver, kidneys, lungs, and longissimus dorsi muscle samples.

**Results:**

Average daily gain and average daily feed intake were reduced (p<0.05) by supplemental Pb during the day 42 to 56. Relative kidney weight to body weight was linearly increased with increasing feeding period in pigs fed the Pb-supplemented diet, but not in pigs fed the control diet (p<0.05). The Pb concentrations in hair, left 5th rib, kidneys, and lungs were linearly increased with longer feeding period in pigs fed the Pb-supplemented diet, but not in pigs fed the control diet (p<0.01).

**Conclusion:**

Dietary Pb supplementation caused growth retardation and Pb accumulation in most organs, particularly in hair, bone, and kidneys in a time-dependent manner.

## INTRODUCTION

Contamination of heavy metals in animal feeds is a problem in animal production and health. Among heavy metals, lead (Pb) exposure in domestic animals due to environmental pollution has been often reported [1]. Contamination of Pb in animal feeds is the major route of Pb exposure in domestic animals [2]. Lead, even at a relatively low concentration, can cause various damages to animals such as poisoning and growth retardation [3,4]. In animals fed with a Pb-contaminated diet, Pb is mainly absorbed through the gastrointestinal tract and accumulated in bone, liver, kidneys, and hair [5,6]. The Pb concentration in a complete diet should not exceed 10 mg/kg in the Republic of Korea, 30 mg/kg in the United States [7] and less than 5 mg/kg in the Europe Union [8]. In addition, Pb accumulated in organs and tissues of animals could be exposed to humans through the food chain [2].

The Pb concentrations in organs and tissues are known to be increased with increasing exposure time or Pb concentration in animal feeds [5]. In addition, Pb can accumulate more in young animals than in old ones [9,10]. However, very limited information is available on dietary Pb toxicity in nursery pigs and on the influence of feeding period of Pb-containing diets to young pigs. Feeding Pb-supplemented diet was hypothesized to cause growth retardation and accumulation of Pb on organs and tissues. Therefore, the objective of this study was to investigate the influence of dietary Pb supplementation and feeding period on growth performance, organ weight, and Pb accumulation in young pigs.

## MATERIALS AND METHODS

### Animal care

The present experiment was reviewed and approved by the Institutional Animal Care and Use Committee of Konkuk University (KU17123).

### Animals, diets, and experimental design

A total of 48 weaned barrows ([Landrace×Yorkshire]×Duroc) with an initial body weight (BW) of 10.4±0.6 kg were used to investigate the influence of dietary Pb supplementation on pigs. The animals were allotted to 2 dietary treatments (supplemental Pb at 0 and 200 mg/kg as Pb acetate) in a completely randomized design using a spreadsheet program developed by Kim and Lindemann [11]. To formulate Pb-supplemented diets, the Pb acetate was supplemented at 366 mg/kg to make 200 mg/kg of Pb (Table 1). Experimental diets were prepared as a 2-phase feeding program (day 0 to 21 and day 21 to 56). The diets were mainly based on corn and soybean meal and were formulated to meet or exceed the nutrient requirement estimates suggested by the NRC [12]. Four pigs were housed in each pen (2.0×2.2 m^2^) that was equipped with a 2-hole feeder and a nipple drinker. Pigs had free access to feed and water throughout the experiment.

### Data and sample collection

Individual BW and feed consumption in each pen were recorded every 14 days (on days 0, 14, 28, 42, and 56) to calculate average daily gain (ADG), average daily feed intake (ADFI), and gain to feed ratio (G:F). Individual feed intake of an animal died on day 2 was estimated using the procedure suggested by Lindemann and Kim [13]. On days 14, 28, 42, and 56, an animal randomly selected from each pen was slaughtered to collect blood samples, hair samples, left 5th rib, heart, liver, kidneys, lungs, and longissimus dorsi muscle (LM) samples. Blood samples were collected from the jugular vein with ethylenediaminetetraacetic acid tubes and stored at 4°C. The organs (heart, liver, kidneys, and lungs) were weighed. The samples except blood were stored in a freezer at −20°C. The relative organ weight to BW was calculated to compensate BW effects.

### Chemical analysis

Diets were finely ground and analyzed for gross energy using a bomb calorimeter (Parr 1261; Parr Instrument Co., Moline, IL, USA). Dry matter (method 930.15), crude protein (method 990.03), ether extract (method 920.39), neutral detergent fiber (method 2002.04), acid detergent fiber (method 973.18), ash (method 942.05), calcium (method 978.02), and phosphorus (method 946.06) in the diet were analyzed as described in AOAC [14]. Diet samples were digested [15] and analyzed for Pb by inductively coupled plasma optical emission spectrometry (Optima 8300; PerkinElmer, Waltham, MA, USA). Lead in blood samples was measured by inductively coupled plasma spectroscopy (Agilent 7900; Agilent Technology, Santa Clara, CA, USA) using a method described by Nunes et al [16]. The left 5th rib, heart, liver, kidneys, lungs, and LM were dried at 105°C using an air-forced drying oven. After drying, samples were finely ground. Before digestion, hair samples were cleaned [17]. The left 5th rib was digested as described by Casteel et al [18]. The samples (hair, heart, liver, kidneys, lungs, and LM) were digested and analyzed following the published procedure [19] with minor modification. Briefly, weighed samples (0.2 g) were placed with 2.5 mL of concentrated HNO_3_ and 0.5 mL of concentrated HCl in a Pyrex glass tube. The tubes packed by a screw cap were kept in a water bath at 85°C for 3 h. After digestion, the tubes were kept at room temperature to cool down, and then the digested solution was filtered through a syringe filter with 0.20 μm of pore diameter. Each sample was diluted to 50 mL with double-distilled water in a volumetric flask. The digested samples were analyzed for Pb by the inductively coupled plasma method (Agilent 7900; Agilent Technology, USA).

### Statistical analysis

Experimental data were analyzed using the MIXED procedure of SAS (SAS Inst. Inc., Cary, NC, USA). Data from a dead pig were excluded in the final calculations and statistical analyses. In the statistical model, only diet was included as the fixed variable for performance data while both diet and feeding period were used as fixed variables for other measurements including organ weight and Pb concentration in organs and tissues. Least square means of each treatment were calculated. Orthogonal polynomial contrasts were used to test the effects of dietary Pb supplementation, feeding periods, and the interaction between dietary Pb supplementation and feeding period. An experimental unit was a pen for growth performance and a pig for organ weight and Pb concentration in organs and tissues [20]. Statistical significance and tendency were determined at p<0.05 and 0.05≤ p<0.10, respectively.

## RESULTS

During the experimental period, all pigs consumed experimental diets well and remained healthy except that one pig in the control group died on day 2.

The ADG, ADFI, or G:F was not affected by supplemental Pb during day 0 to 14 and day 14 to 28 (Table 2). However, pigs fed a diet supplemented with Pb tended to show decreased final BW (p = 0.091), ADG (p = 0.081), and ADFI (p = 0.067) during day 28 to 42 compared with those of the control group. Final BW and ADFI were decreased (p<0.05) by supplemental Pb during day 42 to 56. However, dietary Pb supplementation had no effect on G:F during any period of the experiment.

There was no interaction between dietary Pb supplementation and feeding period for the weight of heart, liver, kidneys, or lungs (Table 3). However, relative kidney weight to BW was linearly increased with increasing feeding period in pigs fed the Pb-supplemented diet but not in pigs fed the control diet (p<0.05).

Supplemental Pb at 200 mg/kg resulted in increased Pb concentrations (p<0.01) in hair, 5th rib, blood, liver, kidneys, lungs, and LM of pigs (Table 4). In pigs fed the Pb-supplemented diet, Pb concentrations in hair, 5th rib, kidneys, and lungs were linearly increased with longer feeding period but not in pigs fed the control diet (p<0.01), indicating the interaction between dietary Pd and linear effects of feeding period. The Pb concentration in liver was quadratically increased with longer feeding period in pigs fed the Pb-supplemented diet, but not in pigs fed the control diet (p<0.01), indicating the interaction between dietary Pd and quadratic effects of feeding period.

When Pb weight in organs were calculated, supplemental Pb resulted in greater (p<0.01) Pb weight in liver, kidneys, and lungs. In the Pb-supplemented group, the Pb weight in liver, kidneys, and lungs were linearly increased with longer feeding period but not in the control group (p<0.05).

## DISCUSSION

The present work revealed that growth performance of pigs fed the Pb-supplemented diet was reduced compared with that of pigs fed the control diet, in agreement with results in previous studies [21,22]. However, some researchers failed to find the negative effects of dietary Pb on growth performance of pigs [23,24]. The lack of responses in performance was likely due to the low concentration of dietary Pb, short experimental period, or both. While dietary Pb concentration was 200 mg/kg in the present work, in the study by Zacharias et al [24], the Pb concentration in the experimental diet was 1.45 mg/kg and feed intake was restricted. The feeding period might be another factor that influences performance responses to dietary Pb. Although the Pb concentration was 250 mg/kg in the study of Reddy et al [23], the feeding period was only 28 days.

The increased kidney weight by dietary Pb supplementation in the present work agrees with results of previous studies using rats and mice [25,26]. Urinary excretion of absorbed Pb is one of major Pb excretion routes, which indicates that kidneys are target organs for Pb toxicity [26]. In rats, supplemental Pb can reduce the concentration of glutathione and antioxidant enzymes [25] but increase cell proliferation in the proximal tubular epithelium of the kidney [27]. These effects of Pb toxicity on the kidney may be a major reason for the increased kidney weight in the Pb-supplemented group observed in the present work.

While the absorption rate of Pb is less than other heavy metals such as copper or mercury, Pb is relatively slowly excreted from animals. Therefore, Pb is accumulated well in most tissues once absorbed into animal body [10]. Previous studies have reported that Pb is highly accumulated in bone, kidneys, liver, and hair [21,22], but not in muscle [24,28], which agrees with the present study.

In Pb toxicity experiments, the Pb concentration and feeding period are important factors. In the present work, only 2 concentrations (0 and 200 mg/kg) of Pb were used, making it impossible to assess dose-dependent polynomial effects. However, 4 feeding periods (14, 28, 42, and 56 days) were employed and time-dependent effects of dietary Pb were observed. In agreement, previous studies reported that Pb concentrations in organs were increased as the feeding period was increased to 84 days in rats [29] and pigs [28]. In rats, Pb concentrations in tissues were increased with increasing Pb intake and the total amount of Pb in tissue did not affect absorption of Pb [9], which indicates that animals perhaps do not regulate Pb absorption or excretion. Although no data are available on Pb absorption or excretion in pigs, the increased Pb concentrations in pig organs and tissues, particularly in bone, hair, and kidneys, by extended feeding of Pb in the present work were likely due to the inability of pigs for excreting absorbed Pb. Lead elimination rate in bone is less than that in other tissues in rats [9] and pigs [28], which explains the greatest Pb accumulation in bone and the linear response of Pb concentration with increasing exposure time in the present work.

The quadratic increase of Pb concentration in organs with longer feeding period may be associated with the age of animals and a dietary milk product. Sharma et al [28] have reported that Pb is more highly accumulated in 30-kg pigs than in 50-kg pigs. Similarly, as the age of rats increased, the absorption of Pb decreased [9,30]. In the present work, Pb was more highly accumulated in pigs likely due to the young age during the first few weeks. Dietary lactose is also a factor that influences the absorption of Pb. In rats, dietary Pb was more highly accumulated in organs when a lactose-added diet was provided compared with a glucose-added diet [30]. Bell and Spickett [31] have also reported that dietary Pb is more efficiently accumulated in rats fed a dried whole milk diet which contains an appreciable amount of lactose than those fed a lactose-hydrolyzed milk diet. In the present work, dried whey containing lactose was included at 10% in the experimental diets during the first 3 weeks. Dietary lactose may be a potential reason for the relatively high Pb concentrations in the day 14 samples of bone, liver, and kidneys of pigs fed a Pb-supplemented diet.

The linear response of Pb weight in the liver, kidneys, and lungs by dietary Pb supplementation is mainly due to the Pb concentrations rather than organ weights that were affected by dietary Pb. The organ Pb weight was calculated by multiplying Pb concentration by organ weight, which represents the amount of Pb accumulated in the organs. The present results indicate that the concentrations of Pb in the organs sufficiently represent the accumulation of Pb in organs.

## CONCLUSION

Dietary Pb supplementation can cause growth retardation and increase kidney weight with an increasing feeding period. In addition, when duration of dietary Pb exposure was increased, the Pb concentration was increased in organs and tissues, although its accumulation rates varied depending on organs and tissues of pigs. Lead was accumulated particularly in hair, bone, and kidneys in a time-dependent manner.

## Figures and Tables

**Table 1:** Ingredient and analyzed chemical compositions of control diets (as-fed basis)

Item	Day 0 to 21	Day 21 to 56
Ingredient (%)		
Ground corn	58.26	62.28
Soybean meal (48% crude protein)	24.00	33.00
Dried whey	10.00	-
Fish meal	3.00	-
Soybean oil	2.00	2.00
L-Lys·HCl (78.8%)	0.38	-
DL-Met (99%)	0.08	-
L-Thr (99%)	0.12	-
Dicalcium phosphate	0.48	1.00
Ground limestone	0.88	0.82
Mineral premix1)	0.25	0.25
Vitamin premix2)	0.25	0.25
Salt	0.30	0.40
Analyzed composition (%)		
Dry matter	90.20	88.80
Gross energy (kcal/kg)	4,025	4,016
Crude protein	19.70	19.10
Ether extract	6.49	6.09
Ash	5.17	4.74
Calcium	0.52	0.52
Phosphorus	0.49	0.34
Neutral detergent fiber	7.51	8.56
Acid detergent fiber	2.39	2.73
Lead3) (Pb, mg/kg)	Not detected	Not detected

1) Provided the following quantities per kg of complete diet: vitamin A, 12,500 IU; vitamin D_3_, 1,000 IU; vitamin E, 125 IU; vitamin K_3_, 6.3 mg; thiamin, 6.3 mg; riboflavin, 25.0 mg; pyridoxine, 12.5 mg; vitamin B_12_, 0.1 mg; pantothenic acid, 100 mg; folic acid, 7.5 mg; niacin, 225 mg; and biotin, 0.5 mg.

2) Provided the following quantities per kg of complete diet: Cu, 87.5 mg as copper sulfate; Fe, 125 mg as iron sulfate; I, 1.0 mg as potassium iodate; Mn, 75 mg as manganese sulfate; Se, 0.25 mg as sodium selenite; and Zn, 60 mg as zinc oxide.

3) Lead acetate was supplemented at 366 mg/kg to the control diets to achieve 200 mg/kg of Pb in the Pb-supplemented diets. The analyzed Pb concentrations were 138.6 and 238.5 mg/kg, respectively, during day 0 to 21 and day 21 to 56.

**Table 2:** Influence of dietary lead (Pb) supplementation on growth performance of pigs1)

Item	Supplemental Pb (mg/kg)		SEM	p-value

	0	200		
Day 0 to 14				
Initial body weight (kg)	10.28	10.49	0.12	0.226
Final body weight (kg)	16.60	16.21	0.42	0.526
Average daily gain (g/d)	451	409	27	0.284
Average daily feed intake (g/d)	736	683	33	0.289
Gain:feed	0.612	0.599	0.022	0.702
Day 14 to 28				
Initial body weight (kg)	16.67	16.30	0.41	0.539
Final body weight (kg)	24.43	23.50	0.73	0.390
Average daily gain (g/d)	554	514	32	0.391
Average daily feed intake (g/d)	1,122	1,046	33	0.139
Gain:feed	0.494	0.490	0.020	0.907
Day 28 to 42				
Initial body weight (kg)	24.88	23.60	0.81	0.289
Final body weight (kg)	35.08	31.22	1.46	0.091
Average daily gain (g/d)	729	545	67	0.081
Average daily feed intake (g/d)	1,510	1,298	73	0.067
Gain:feed	0.480	0.406	0.038	0.199
Day 42 to 56				
Initial body weight (kg)	34.92	30.58	1.34	0.046
Final body weight (kg)	44.83	39.83	1.07	0.008
Average daily gain (g/d)	708	661	58	0.572
Average daily feed intake (g/d)	2,144	1,626	136	0.022
Gain:feed	0.339	0.429	0.061	0.325

SEM, standard error of the means.

1) Each least squares mean represents 6 replicated pens; a pig selected from each pen was slaughtered at the end of each 14-day period.

**Table 3:** Influence of dietary lead (Pb) supplementation and feeding period on organ weight of pigs (wet basis)

Item	Pb (mg/kg): Period (d):	0				200				SEM	p-value1)				
		
		14	28	42	56	14	28	42	56		Pb	L	Q	Pb×L	Pb×Q
No. of observations		6	5	6	6	6	6	6	6						
Organ weight (g)															
Heart		88	129	170	210	87	127	145	189	9	0.048	<0.001	0.952	0.133	0.816
Liver		479	627	887	1,033	422	580	744	923	40	0.002	<0.001	0.867	0.295	0.836
Kidneys		81	116	173	219	81	134	180	254	10	0.035	<0.001	0.247	0.127	0.691
Lungs		193	259	353	448	196	303	344	399	34	0.904	<0.001	0.805	0.323	0.395
Organ weight relative to body weight2) (%)															
Heart		0.54	0.56	0.49	0.47	0.54	0.55	0.45	0.47	0.02	0.583	<0.001	0.661	0.800	0.317
Liver		2.92	2.72	2.52	2.30	2.65	2.52	2.36	2.31	0.10	0.028	<0.001	0.800	0.142	0.715
Kidneys		0.49	0.50	0.49	0.49	0.51	0.58	0.57	0.64	0.03	<0.001	0.074	0.880	0.037	0.856
Lungs		1.18	1.14	1.01	1.00	1.23	1.33	1.08	1.00	0.10	0.246	0.010	0.617	0.664	0.470

SEM, standard error of the means.

1) Pb, dietary Pb supplementation; L, linear effect of feeding period; Q, quadratic effect of feeding period; Pb×L, interaction between dietary Pb supplementation and linear effect of feeding period; Pb×Q, interaction between dietary Pb supplementation and quadratic effect of feeding period.

2) Relative organ weights to body weight (%) = organ weight (kg)/body weight of pig (kg)×100.

**Table 4:** Influence of dietary lead (Pb) supplementation and feeding period on Pb concentration of pig organs (wet basis except for hair)

Item	Pb (mg/kg):	0				200				SEM	p-value1)				
		
	Period (d):	14	28	42	56	14	28	42	56		Pb	L	Q	Pb×L	Pb×Q
No. of observations		6	5	6	6	6	6	6	6						
Hair (mg/kg)		1.42	0.92	1.47	1.36	4.35	9.15	17.63	26.23	2.06	<0.001	<0.001	0.460	<0.001	0.547
Left 5th rib (mg/kg)		3.58	1.64	1.68	1.90	44.58	42.68	100.58	149.03	9.35	<0.001	<0.001	0.046	<0.001	0.066
Blood (μg/dL)		0.013	0.007	0.003	0.003	0.378	0.582	0.454	0.521	0.031	<0.001	0.178	0.059	0.089	0.042
Pb concentration in fresh tissue2) (mg/kg)															
Heart		0.60	0.70	0.92	0.59	0.81	0.83	0.56	1.19	0.17	0.206	0.286	0.711	0.512	0.027
Liver		0.27	0.49	0.65	1.06	10.49	6.99	8.64	12.14	0.74	<0.001	0.047	0.001	0.368	0.002
Kidneys		0.50	0.23	0.17	0.20	7.95	7.03	8.89	10.09	0.44	<0.001	0.008	0.049	0.001	0.132
Lungs		0.21	0.10	0.10	0.07	0.27	0.31	0.37	0.36	0.04	<0.001	0.780	0.857	0.002	0.245
LM		0.14	0.20	0.13	0.24	0.30	0.26	0.27	0.25	0.03	<0.001	0.663	0.437	0.100	0.715
Pb weight in fresh organ (mg)															
Heart		0.05	0.09	0.16	0.12	0.07	0.11	0.09	0.23	0.03	0.433	<0.001	0.675	0.305	0.026
Liver		0.13	0.31	0.57	1.11	4.46	4.04	6.60	11.27	0.61	<0.001	<0.001	0.002	<0.001	0.007
Kidneys		0.04	0.03	0.03	0.04	0.64	0.94	1.65	2.59	0.13	<0.001	<0.001	0.065	<0.001	0.087
Lungs		0.04	0.03	0.04	0.03	0.05	0.09	0.15	0.15	0.02	<0.001	0.022	0.559	0.012	0.444

LM, longissimus dorsi muscle; SEM, standard error of the means.

1) Pb, dietary Pb supplementation; L, linear effect of feeding period; Q, quadratic effect of feeding period; Pb×L, interaction between dietary Pb supplementation and linear effect of feeding period; Pb×Q, interaction between dietary Pb supplementation and quadratic effect of feeding period.

2) The values were calculated based on the Pb concentrations in dried organ and moisture concentration in fresh organ.
